# Caregivers' Attitudes Toward COVID-19 Vaccination in Children and Adolescents With a History of SARS-CoV-2 Infection

**DOI:** 10.3389/fped.2022.867968

**Published:** 2022-04-07

**Authors:** Danilo Buonsenso, Piero Valentini, Marina Macchi, Francesco Folino, Carola Pensabene, Maria Francesca Patria, Carlo Agostoni, Silvana Castaldi, Maurizio Lecce, Maria Lorella Giannì, Paola Marchisio, Gregorio P. Milani, The CHICO Study Group

**Affiliations:** ^1^Department of Woman and Child Health and Public Health, Fondazione Policlinico Universitario “A. Gemelli”, Rome, Italy; ^2^Center for Global Health Research and Studies, Dipartimento di Scienze Biotecnologiche di Base, Cliniche Intensivologiche e Perioperatorie, Università Cattolica del Sacro Cuore, Rome, Italy; ^3^Department of Clinical Science and Community Health, University of Milan, Milan, Italy; ^4^Fondazione IRCCS Ca' Granda Ospedale Maggiore Policlinico, Pediatric Unit, Milan, Italy; ^5^Department of Biomedical Sciences for Health, Postgraduate School of Public Health, University of Milan, Milan, Italy; ^6^Fondazione IRCCS Ca' Granda Ospedale Maggiore Policlinico, Milan, Italy; ^7^Neonatal Intensive Care Unit, Fondazione IRCCS Cà Granda Ospedale Maggiore Policlinico, Milan, Italy; ^8^Department of Pathophysiology and Transplantation, University of Milan, Milan, Italy

**Keywords:** hesitancy, pediatrics, parental, caregivers, vaccination, influenza, papillomavirus, long covid

## Abstract

**Background:**

Limited data are available on the attitudes of caregivers toward COVID-19 vaccination in children and adolescents with a history of SARS-CoV-2 infection or Long Covid symptoms. The aim of this study was to investigate the vaccine hesitancy among caregivers of children and adolescents with a documented history of SARS-CoV-2 infection and to explore the possible associations between COVID-19 manifestations and the acceptance of the vaccine.

**Methods:**

Caregivers of children or adolescents with a microbiologically confirmed diagnosis of SARS-CoV-2 infection evaluated in two University Hospitals were interviewed.

**Results:**

We were able to contact 132 caregivers and 9 declined to participate. 68 caregivers (56%) were in favor of COVID-19 vaccination for their child. In the multiple logistic regression, child's age (OR 1.17, 95%CI 1.06–1.28) and hospitalization due to COVID-19 (OR 3.25, 95%CI 1.06–9.95) were positively associated with being in favor of COVID-19 vaccination. On the contrary, the occurrence of child's Long Covid was associated with a higher likelihood of being against the vaccination (OR 0.28, 95%CI 0.10–0.80).

**Conclusions:**

This preliminary study shows that only about half of the interviewed parents of children and adolescents with a previous SARS-CoV-2 infection are willing to vaccinate them to prevent a repeated COVID-19 infection. These findings might help healthcare workers to provide tailored information to caregivers of children with a previous SARS-CoV-2 infection.

## Introduction

The immunization against Coronavirus Disease 2019 (COVID-19) is considered one of the key public measures to combat the pandemic and the most effective tool to prevent symptomatic or severe disease (1–3). Recent trials showed the efficacy and safety of BNT162b2 Covid-19 mRNA vaccine in children aged 5 years and older (4, 5).

Despite the increasing production and availability of the vaccine for children, several data point out that many caregivers are hesitant toward COVID-19 vaccination of their children (6–8). Previous studies have identified that many factors might underlie the refusal of pediatric COVID-19 vaccine including, among others, the perception of a low risk of SARS-CoV-2 infection and complications in children (9, 10). However, the spread of the virus in the worldwide population is leading to a growing number of children affected by SARS-CoV-2 and some of them require hospitalization or develop complications such as Long Covid (1, 11, 12). Moreover, a subgroup of children might develop rare but severe complications, such as the Multisystem Inflammatory Syndrome (MIS-C) which often requires intensive care admission (13). Importantly, two studies from France and United States provided preliminary evidence that COVID-19 vaccinations might also prevent the development of MIS-C (14, 15).

Since children, similarly to adults, might be re-infected by new variants of SARS-CoV-2 and the virus spread in the pediatric population is growing (16, 17), children eligible for vaccination and with a history of SARS-CoV-2 infection are likely to increase more and more over time. The Italian Society of Pediatrics currently suggests that children with previous infection should receive a regular vaccination schedule starting three to 6 months after the infection (18). Moreover, other social reasons may, at least theoretically, induce parents to vaccinate their children. For example, school closures have been implemented worldwide as a non-pharmaceutical approach to prevent the spread of the virus within the community, but detrimental consequences on children' physical and mental health have been observed (19–21). Therefore, high vaccinations rates in children and teachers might further support a return to a normal school attendance.

However, very limited data are available on the attitudes of caregivers toward COVID-19 vaccination in children with a history of SARS-CoV-2 infection or Long Covid. The aim of this study was to investigate the vaccine hesitancy among caregivers of children and adolescents with a documented history of SARS-CoV-2 infection and to explore the possible associations between COVID-19 manifestations and the acceptance of the vaccine.

## Methods

The study was conducted between November 01, 2021, and January 15, 2022. Eligible for the study were subjects admitted to the Pediatric emergency department (ED) of the Fondazione Ca' Granda Ospedale Maggiore Policlinico, Milan and the Fondazione Policlinico Universitario A. Gemelli, Rome, Italy between July 01, 2020, and June 31, 2021, with a diagnosis of SARS-CoV-2 infection, ascertained by a molecular test in the ED.

The caregivers were contacted by phone and were asked to answer a structured interview. The survey was composed by five sections and investigated the following items: 1) demographic data and educational level of the caregivers; 2) demographic data, the presence of chronic diseases, the manifestation of COVID-19 including the presence of symptoms, need for hospitalization or intensive care, and the development of Long COVID symptoms [defined as having persisting symptoms such as dyspnea, mental confusion, fatigue; chest pain, problems associated to speech, anxiety and altered mood, muscular pain, fever, loss of taste and smell - never reported before COVID-19—for at least 12 weeks (22)] of the child; 3) COVID-19 history in first-degree relatives including the need of hospitalization or intensive care, and the occurrence of death; 4) the caregiver attitudes toward COVID-19 vaccination, if they changed opinion about COVID-19 vaccination after the SARS-CoV-2 infection of their child and his/her opinion on other vaccinations (against Papillomavirus and *Streptococcus pneumoniae*). Finally, if the caregiver and/or the child received at least one dose of influenza vaccine in the previous 3 years was also investigated. The questionnaire is reported within the online Supplementary Material.

Before administration, the survey was pilot tested among 5 caregivers and 5 physicians.

### Data Management and Statistical Analysis

All data were anonymously collected in a predefined, online database. Response rate was calculated as the number of subjects who accepted to participate divided by the number of subjects who participated plus those who declined. Continuous and categorical data are presented as median and interquartile range (IQR) or absolute frequency and percentage, respectively. Caregivers who were willing to vaccinate their children against COVID-19 or whose children had been already vaccinated were collapsed into the category “In favor of COVID-19 vaccination”, whereas subjects in doubt or against the vaccination of their children against COVID-19 were collapsed into the category “Others”. Wilcoxon rank sum test was used to compare continuous data of the above-mentioned two groups and the Fisher's exact test or Chi-squared test to compare categorical data, as appropriate. Furthermore, a logistic regression including being “In favor of COVID-19 vaccination” as dependent variable and the caregiver and child's sex, the educational level of the caregiver, the number of months since the child had the COVID-19, the presence of chronic diseases or Long Covid in the child, the need for child's admission for COVID-19, history of a first-degree relative who needed admission due to COVID-19, was performed. The Akaike information criterion was applied to select the best multiple model. Significance was assumed for a *p*-value < 0.05. Analyses were performed using the open-source statistical software R, Vienna, version 3.5.3 (11 March 2019). The study was approved by the institutional ethics committee and consent was obtained before the interview from all participants.

## Results

We were able to contact a total of 132 caregivers and 11 declined to participate. The median age of the caregivers who accepted to participate was 42 [IQR 38–47] years (males = 24, 20%), Table 1. The majority had a university or post university degree (*N* = 66, 54%). The median age of their child was 9.0 [2.0–12.0] years (males = 73, 60%). Most respondents (*N* = 103, 85%) had at least one relative with a history of COVID-19. The majority wanted to vaccinate or had already vaccinated their child against Papillomavirus (N=78, 64%) or *Streptococcus pneumoniae* (*N* = 89, 74%).

**Table 1:** Baseline characteristics of the caregivers and children.

**N**	**121**
**Caregiver**	
Age, years	42 [38–47]
Sex, female	97 (80)
Educational level	
Secondary school	19 (16)
High School	36 (30)
University	43 (35)
Post University	23 (19)
Influenza vaccination in the last 3 years, yes	35 (29)
**Child**	
Age, years	9.0 [2.0–12.0]
Sex, female	48 (40)
Underlying chronic disease, yes	18 (15)
Influenza vaccination in the last 3 years, yes	23 (19)
Months from COVID-19 infection	11 [6–13]
Symptomatic COVID-19, yes	89 (74)
Hospitalization due to COVID-19, yes	27 (22)
Need of intensive care due to COVID-19, yes	0 (0.0)
Long Covid symptoms, yes	45 (37)
**COVID19 history in first-degree relatives**	
History of COVID-19, yes	103 (85)
Hospitalization due to COVID-19, yes	17 (14)
Need of intensive care due to COVID-19, yes	8 (6.5)
Death due to COVID-19, yes	4 (3.3)
Long Covid symtoms, yes	46 (38)
**Caregiver's opinion on other vaccinations**	
Vaccination against papillomavirus	
I want my child to receive this vaccination/ My child has been already vaccinated	78 (64)
I know this infection, but I am unsure/I do not want my child to receive the vaccination	25 (21)
I do not know the disease	18 (15)
Vaccination against *Streptococcus pneumoniae*	
I want my child to receive this vaccination/ My child has been already vaccinated	89 (74)
I know this infection, but I am unsure/I do not want my child to receive the vaccination	15 (12)
I do not know the disease	17 (14)

*Data are given as median and interquartile range or absolute frequency and percentage*.

**Figure 1:**
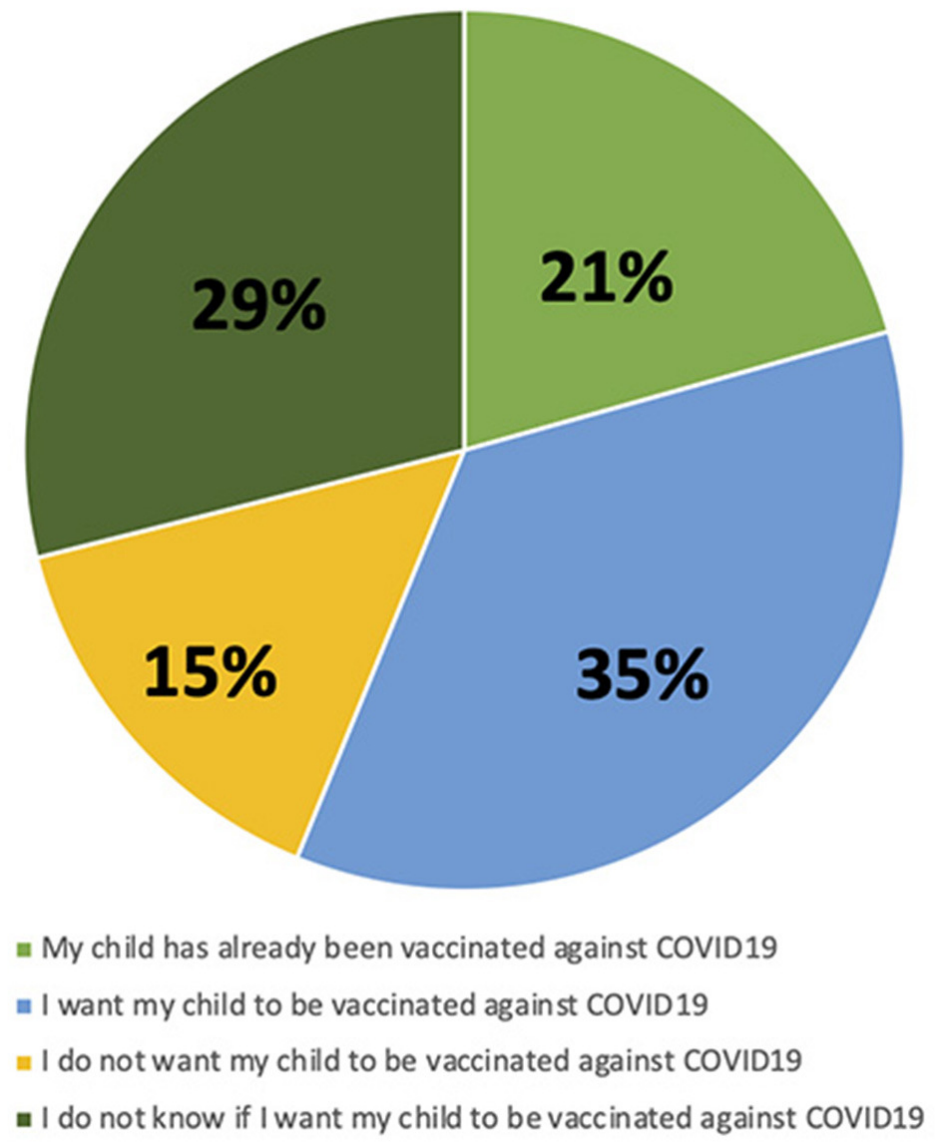
Caregivers' attitudes toward COVID-19 vaccination of his/her child.

**Figure 2:**
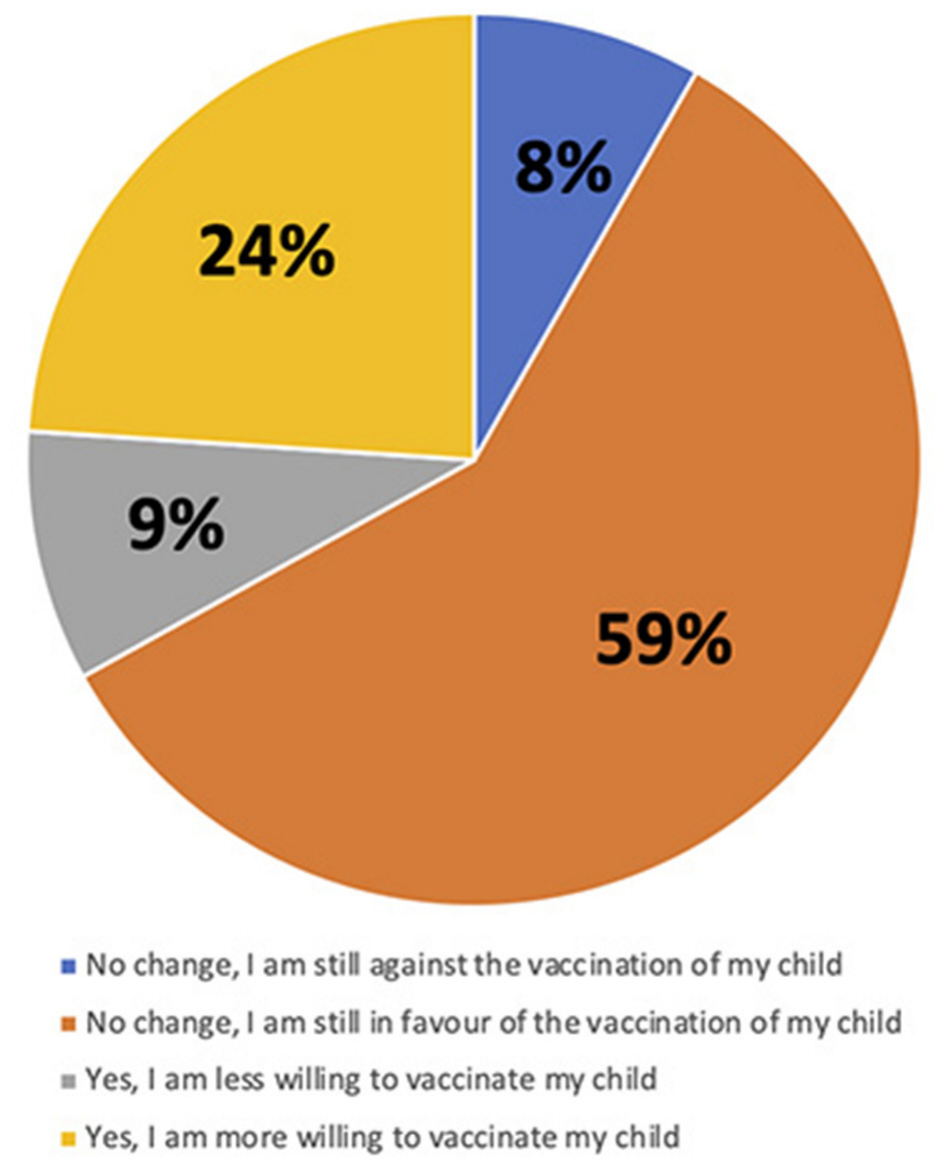
Change of caregivers' attitudes toward COVID-19 vaccination of his/her child after the child's COVID-19 infection.

Most caregivers (*N* = 68, 56%) were in favor of COVID-19 vaccination for their child (Figure 1). A total of 81 (67%) caregivers stated that they have not changed their opinion about COVID-19 vaccination: 71 of them kept their willingness to vaccinate their child and 10 remain against to COVID-19 vaccination (Figure 2). On the contrary, 40 (33%) of the caregivers changed their opinion about COVID-19 vaccination after the SARS-CoV2- infection of their child: 29 became in favor and 11 against the vaccination.

Table 2 reports the characteristics of the caregivers in favor of COVID-19 vaccination as compared to the others (univariate analysis). The only differences between these two groups were the older age of caregivers (44 [40-49] vs. 40 [36-46] years, *p* = 0.008) and of the child (10.5 [4.0–14] vs. 6.0 [2.0–10] years, *p* = 0.001) in the group in favor of the vaccination as compared to the others. In the multiple logistic regression, child's age (OR 1.17, 95%CI 1.06–1.28) and hospitalization due to COVID-19 (OR 3.25, 95%CI 1.06–9.95) were positively associated with being in favor of COVID-19 vaccination. On the contrary, the occurrence of child's Long Covid symptoms was inversely associated (OR 0.28, 95%CI 0.10–0.80) with being in favor of COVID-19 vaccination (Table 3).

**Table 2:** Comparison of the characteristics of caregivers in favor of COVID19 vaccination for their children and the other caregivers.

	**In favor**	**Others**	***P***
**N**	68	53	
**Caregiver**			
Age, years	44 [40–49]	40 [36–46]	**0.008**
Sex, female	51 (75)	46 (87)	0.12
Educational level			
Secondary school	8 (12)	11 (20)	0.35
High School	23 (34)	13 (25)	
University	26 (38)	17 (32)	
Post University	11 (16)	12 (23)	
Influenza vaccination in the last 3 years, yes	19 (28)	16 (30)	0.84
**Child**			
Age, years	10.5 [4.0–14]	6.0 [2.0–10]	**0.001**
Sex, female	24 (35)	24 (45)	0.35
Underlying chronic disease, yes	9 (13.2)	9 (17)	0.61
Influenza vaccination in the last 3 years, yes	11 (16)	12 (23)	0.48
Months from COVID19 infection	12 [7–13]	8 [5–12]	0.15
Long Covid, yes	23 (34)	22 (42)	0.26
Symptomatic Covid, yes	48 (71)	41 (77)	0.53
Hospitalization due COVID19, yes	18 (26)	9 (17)	0.27
**COVID19 history in first-degree relatives**			
History of COVID19, yes	55 (81)	48 (91)	0.19
Hospitalization due to COVID19, yes	10 (16)	7 (14)	0.79
Need of intensive care due to COVID19, yes	4 (8.9)	4 (9.5)	1.00
Death due to COVID19, yes	1 (2.4)	3 (7.5)	0.36
Long Covid symptoms, yes	23 (41)	23 (52)	0.31
**Caregiver's opinion on other vaccinations**			
Vaccination against papillomavirus			
I want my child to receive this vaccination/ My child has been already vaccinated	49 (72)	29 (55)	0.06
I know this infection, but I am unsure/I do not want my child to receive the vaccination	9 (13)	16 (30)	
I do not know the disease	10 (15)	8 (15)	
Vaccination against Streptococcus pneumoniae			
I want my child to receive this vaccination/ My child has been already vaccinated	49 (71)	40 (75)	0.96
I know this infection, but I am unsure/I do not want my child to receive the vaccination	9 (13)	6 (11)	
I do not know the disease	10 (15)	7 (13)	

*Data are given as median and interquartile range or absolute frequency and percentage. Significant p-values (i.e. p-values < 0.05) are in bold*.

**Table 3:** Results of the logistic regression.

	**OR**	**Lower 95%CI**	**Upper 95%CI**	**P**
Child's age	1.17	1.06	1.28	**0.001**
Child's sex	2.48	0.98	6.26	0.06
Presence of child's chronic disease	0.34	0.09	1.26	0.11
Child's Long Covid symptoms	0.28	0.10	0.80	**0.017**
Child's hospitalization due to COVID-19	3.25	1.06	9.95	**0.039**

*The favor towards COVID-19 vaccination of the child was considered as dependent variable. The following independent variables were included: the caregiver and child's sex, the educational level of the caregiver, the child's age, the months since the child had the COVID-19, the presence of chronic diseases or Long Covid symptoms in the child, the hospitalization of the child for COVID-19, history of a parent who needed admission due to COVID-19. The Akaike information criteria was applied to select the best model. Significant p-values (i.e. p-values < 0.05) are in bold*.

## Discussion

In this study, we have assessed parents' attitudes toward COVID-19 vaccination in children and adolescents with a previous SARS-CoV-2 infection. Overall, we found that <60% of the interviewed parents were willing to vaccinate their children. Interestingly, parents of children who experienced long Covid symtpoms, were more likely to be in favor of the vaccination, while those of children experience Long Covid were more frequently against the vaccination. To our knowledge, vaccine hesitancy among caregivers of children with a documented history of SARS-CoV-2 infection has never been explored so far, and our findings may support the development of personalized communication strategies, which will be crucial in the next months since a huge proportion of children with previous SARS-CoV-2 infection are now eligible for vaccination. This is a critical area for public health interventions since subjects infected during the first waves of the pandemic might not be protected against infection with new variants, including Omicron (23).

A significant association was found between being in favor of vaccination and having had a child previously hospitalized with COVID-19. These findings could be expected since parents that experienced the more severe spectrum of pediatric COVID-19 might be more worried of a new infection. During the first wave of the pandemic, early data from China (24), Italy (25, 26) and Europe (27) showed that children were mostly spared from COVID-19 and only a minority developed a complicated disease. These findings, along with the severe clinical picture and high mortality rates in adults, translated to the public misconception that COVID-19 was not a serious danger for children and that known short term complications, including myocarditis, and unknown long-term effects, could not justify the benefit of preventing COVID-19 by a vaccination (28). However, during the following waves of the pandemic, increasing evidence showed that not only COVID-19 can be severe in childhood, but children can also develop the MIS-C (29). Since COVID-19 vaccination might also prevent MIS-C (14), it is expected that parents of children who experienced severe COVID-19 might prefer to avoid a new infection and its possible acute complications. Accordingly, our data suggest that better communication strategies should be developed to successfully inform the public opinion that children can have severe complications from SARS-CoV-2 infections, leading to a higher adherence to the vaccination campaign for previously infected children. In this regard, the role of family pediatricians (or general practitioners) in appropriately informing parents is pivotal since they usually have a close and long-lasting relationship with the family.

Conversely, the finding that parents of children and adolescents with Long Covid symptoms are more likely against vaccination is unexpected and might rely on a complex scenario. So far, the real burden of pediatric Long Covid is still unclear (22), however there is recognition from independent studies that a subset of children may experience it (30, 31). Although it is still unclear if COVID-19 vaccination can prevent Long Covid (32), it is possible to speculate that preventing the infection might also prevent its consequences. A recent study from Israel found that vaccinated adults were at lower risk of developing Long Covid, even after a breakthrough infection (33). Therefore, since parents of children with history of Long Covid are aware of the impact that long-lasting symptoms may have on a child's routine, we would have expected a more propension toward the vaccination to prevent a new infection and a Long Covid relapse. On the other hand, it is possible that these parents can be worried that an immune stimulation can relapse the symptoms and make their child's routine worse. However, preliminary data showed that a group of Long Covid patients had their symptoms improved after vaccination (34), leading to the hypothesis that vaccination itself might become a part of Long Covid management. The rationale behind this is that vaccines might re-equilibrate immune responses or help the organism in viral clearance or divert autoimmune lymphocytes through innate cytokines (35). Overall, uncertainties on the pathogenesis of Long Covid, along with the possible effects of vaccination on improvement or worsening of symptoms, may justify parents' fears for vaccination of their children with Long Covid. Since this is a delicate point of public health interventions, our findings can be used to develop appropriate public health strategies including the development of new studies on the relationship between vaccination and Long Covid in children.

In Italy, similarly to the other European countries, a priority for vaccination was given to subjects at higher risk of worse outcomes (e.g., elderly) and those more at risk of infection (e.g., healthcare workers) (36). In December 2021, after the approval of COVID-19 vaccination in children and adolescents, the vaccine has been offered to these subjects in dedicated healthcare structures. Although Italian family doctors have not been involved in children vaccination so far, they might play a key-function in the parental counseling. Their role, which was not investigated in the current survey, deserves consideration in future studies.

The results of our study should be also analyzed in view of the rapidly changing pandemic scenario. Very recent data showed that hybrid immunity is stronger than natural immunity (37). Therefore, it is possible that many participants of this study believed that natural immunity sufficed to prevent a new infection. Similarly, the caregivers' perception of the severity of Omicron infection might have modified during the study period. When this variant appeared media from around the world reported that an increasing number of infected children had been hospitalizing (38) and it was mainly due to a higher severity of disease (39). Later, a few studies have argued against such hypotheses (40, 41). These changing scenarios may have affected parents' responses, but our sample was not large enough to address this potential variability.

Our study has limitations. First, our preliminary study involved a relatively low number of participants and it could not be planned to analyze the possible role of MIS-C development on the vaccine acceptance. Second, data were collected from two main centers in Center and North Italy, while no centers from Southern Italy have been included. Given cultural differences along the country, our data might not be extrapolated to different areas. Third, we have not analyzed in depth the reasons behind a specific family position, nor we have investigated the children' perspective. Mostly, our study does not provide a response to the question “why parents want, or do not, their children to be vaccinated against COVID-19?” Future multicenter studies addressing this question are necessary to support transnational efforts to increase vaccine confidence (42). Fourth, most collected information was based on parents' reporting on not ascertained on medical records. Finally, we did not investigate if the caregivers were infected by COVID-19 and the possible manifestations of such infection. The evaluation of the possible role of previous COVID-19 experience in both children and relatives with and without Long Covid, represents a strength of our study.

In conclusion, our study shows that only about half of the interviewed parents of children and adolescents with a previous SARS-CoV-2 infection are willing to vaccinate them against COVID-19. Parents were more in favor of vaccination if their children were hospitalized for COVID-19, but less if children had experienced Long Covid symptoms. These findings might help healthcare workers to provide tailored information to caregivers of children and adolescents with a previous SARS-CoV-2 infection. Finally, this study and the evolving scenario of this pandemic point out that new international studies addressing the reasons behind parents' attitudes toward COVID-19 vaccine are necessary.

## Data Availability Statement

The raw data are available upon resonable request to the corresponding author.

## Ethics Statement

The studies involving human participants were reviewed and approved by Comitato Etico | Policlinico Agostino Gemelli Roma. The patients/participants provided their informed consent to participate in this study.

## Author Contributions

DB, MFP, CA, SC, ML, MLG, PM, and GM conceptualized and designed the study. PV, MM, FF, and CP collected the data. GM performed the statistical analyses. DB and GM draft the first version of the manuscript. All authors gave a significant contribution to data interpretation in their field of expertise. All authors contributed to the article and approved the submitted version.

## Funding

This study was partially funded by a grant of the Italian Ministry of Health ricerca Corrente 2020.

## Conflict of Interest

The authors declare that the research was conducted in the absence of any commercial or financial relationships that could be construed as a potential conflict of interest.

## Publisher's Note

All claims expressed in this article are solely those of the authors and do not necessarily represent those of their affiliated organizations, or those of the publisher, the editors and the reviewers. Any product that may be evaluated in this article, or claim that may be made by its manufacturer, is not guaranteed or endorsed by the publisher.
