# Supplementary material for: Caregivers' Attitudes Toward COVID-19 Vaccination in Children and Adolescents With a History of SARS-CoV-2 Infection
Source: Front Pediatr. 2022 Apr 7;10:867968. doi: 10.3389/fped.2022.867968 (PMC9021633; doi:10.3389/fped.2022.867968)
Supplement: Supplementary file 1 [file Data_Sheet_1.pdf]

Supplementary material

Caregivers disposition towards COVID-19 vaccination in children with a history of SARS-CoV-2 infection or Long Covid

Questionnaires employed for the survey

1) Age of the caregivers

2) Sex of the caregivers

- Female
- Male

3) Educational level of the caregivers

- Primary school
- Secondary school
- University
- Post University

4) Age of the child

5) Sex of the child

- Female
- Male

6) Does your child present any chronic disease?

- Yes
- No

7) How many months have passed since the SARS-CoV-2 infection of your child?

8) Did your child present a symptomatic SARS-CoV-2 infection?

- Yes
- No

9) Did your child require hospitalization due to the SARS-CoV-2 infection?

- Yes
- No

10) Did your child require intensive care due to the SARS-CoV-2 infection?

- Yes
- No

11) Have your child presented symptoms of Long Covid (i.e. persisting symptoms such as dyspnea, mental confusion, fatigue; chest pain, problems associated to speech, anxiety and altered mood, muscular pain, fever, loss of taste and smell - never reported before SARS-CoV-2 infection- for at least 12 weeks)?

- Yes
- No

12) Did any of your first-degree relatives present a SARS-CoV-2 infection (documented by a molecular diagnosis)?

- Yes
- No

13) Did any of your first-degree relatives need for hospitalization due to a SARS-CoV-2 infection?
- Yes
- No

14) Did any of your first-degree relatives need for intensive care due to a SARS-CoV-2 infection?
- Yes
- No

15) Did any of your first-degree relatives die due to a SARS-CoV-2 infection?
- Yes
- No

16) Have any of your first-degree relatives presented symptoms of Long Covid (i.e. persisting symptoms such as dyspnea, mental confusion, fatigue; chest pain, problems associated to speech, anxiety and altered mood, muscular pain, fever, loss of taste and smell - never reported before SARS-CoV-2 infection- for at least 12 weeks)?
- Yes
- No

17) What do you think about COVID-19 vaccination of your child?
- I am willing to vaccinate my child
- My child has already been vaccinated
- I do not want my child to be vaccinated
- I do not know if I want my child to be vaccinated against COVID-19

18) What do you think about the vaccination of your child against Papillomavirus infection?
- I am willing to vaccinate my child/my child has already been vaccinated
- I know this infection, but I am unsure/I do not want my child to receive the vaccination
- I do not know the disease

19) What do you think about the vaccination of your child against Papillomavirus infection?
- I am willing to vaccinate my child/my child has already been vaccinated
- I know this infection, but I am unsure/I do not want my child to receive the vaccination
- I do not know the disease

20) Have you changed your disposition towards COVID-19 vaccination your child after his/her SARS-CoV-2 infection?
- No, I am still in favor of the vaccination of my child
- No, I am still against the vaccination of my child
- Yes, I am more willing to vaccinate my child
- Yes, I am less willing to vaccinate my child

21) Did you receive a dose of vaccination against influenza in the last 3 years (2018, 2019 and 2020)?
- Yes
- No

22) Did your child receive a dose of vaccination against influenza in the last 3 years (2018, 2019 and 2020)?
- Yes
- No
